# Genetic characterization of porcine parainfluenza virus 1 (PPIV-1) in pig farms: first report of PPIV-1 in Thailand and Myanmar

**DOI:** 10.3389/fvets.2025.1435920

**Published:** 2025-02-19

**Authors:** Hnin Wai Phyu, Kamonpan Charoenkul, Chanakarn Nasamran, Kitikhun Udom, Eaint Min Phyu, Yu Nandi Thaw, Han Win Soe, Supassama Chaiyawong, Thant Nyi Lin, Min Thein Maw, Alongkorn Amonsin

**Affiliations:** ^1^Center of Excellence Emerging and Re-emerging Infectious Diseases in Animals (CUEIDAs), Chulalongkorn University, Bangkok, Thailand; ^2^Department of Veterinary Public Health, Faculty of Veterinary Science, Chulalongkorn University, Bangkok, Thailand; ^3^Department of Veterinary Public Health, University of Veterinary Science, Nay Pyi Taw, Myanmar; ^4^Livestock Breeding and Veterinary Department, Ministry of Agriculture, Livestock and Irrigation, Nay Pyi Taw, Myanmar

**Keywords:** genetic characterization, Myanmar, pig, PPIV-1, Thailand

## Abstract

Porcine parainfluenza virus 1 (PPIV-1) is a paramyxovirus causing respiratory infections in pigs and has been reported worldwide. In this study, we conducted a cross-sectional survey of PPIV-1 in pig farms in Thailand and Myanmar from January 2022 to December 2023. Nasal swab samples from pigs in Thailand (*n* = 1,042) and Myanmar (*n* = 449) were collected from clinically healthy pigs and pigs with respiratory signs. PPIV-1 detection was carried out using the L gene-specific RT-PCR assay. Our results showed that 3.65% (38/1042) and 7.57% (34/449) were positive for PPIV-1 in Thailand and Myanmar, respectively. The viruses (*n* = 15) were subjected to whole genome sequencing (*n* = 4) and F and HN gene sequencing (*n* = 11). Genetic and phylogenetic analyses showed that Thai PPIV-1 (*n* = 7) was grouped into PPIV-1 lineage II (American lineage) and closely related to American and Chinese strains. On the other hand, one Thai PPIV-1 strain (*n* = 1) and Myanmar PPIV-1 (*n* = 7) belonged to lineage I (European lineage) and was closely related to European, Hong Kong (China), and South Korean strains. Our findings suggest that PPIV-1s from both lineages (I and II) are circulating in pigs in Thailand, and PPIV-1 of lineage I is circulating in pigs in Myanmar, suggesting high genetic diversity of PPIV-1 in the Southeast Asia region. This study is the first to report whole-genome sequences of PPIV-1 from pigs in Thailand and Myanmar. Our result provided insights and information about the current disease status and genetic diversity of PPIV-1 in pig farms, which will benefit further animal disease surveillance, prevention, and control.

## Introduction

Porcine parainfluenza virus 1 (PPIV-1), also known as porcine respirovirus 1 (PRV1), is a paramyxovirus that causes respiratory infections in pigs, first isolated in 2013. The PPIV-1 is an enveloped, negative-sense, single-stranded, non-segmented RNA virus within the family *Paramyxoviridae*, the genus *Respirovirus*. The genome size of PPIV-1 is around 15 kb. The viral genome consists of six open reading frames (ORFs) that encode the structural proteins, including nucleocapsid (N), phosphoprotein (P), matrix (M), fusion (F), haemagglutinin-neuraminidase (HN) and large (L) proteins (3’-N-P-M-F-HN-L-5′) (1, 2). Two significant surface glycoproteins, F and HN, are crucial in viral fusion between the viral envelope and cell membrane. They also play a role in viral attachment, entry into the host cells, and release from the cells (3, 4). F and HN glycoproteins are used for molecular epidemiological study of PPIV-1 (2). Based on F gene analysis, PPIV-1 can be classified into two distinct genetic lineages: lineage I (European lineage) and lineage II (American lineage) (5). The HN gene analysis also provided phylogenetic clustering similar to the results observed by the F gene and complete genome analysis (6).

PPIV-1 was first reported in nasopharyngeal and rectal swabs collected from slaughtered pigs in Hong Kong, China, 2009 and was first isolated in 2013. The genome of PPIV-1 was found to be closely related to human parainfluenza virus 1 (HPIV-1) and Sendai virus (SeV), which suggests that the virus has zoonotic potential (1). However, this speculation about the zoonotic potential of PPIV-1 was reported without experimental evidence of cross-species transmission of human infection. PPIV-1 has been distributed widely in pig farms across many countries, including Brazil, Chile, Germany, Netherlands, Italy, Poland, South Korea, and the USA (6–13). PPIV-1 has been isolated in pigs without or with respiratory symptoms such as coughing, sneezing, and nasal discharge (1, 10). The potential impact of PPIV-1 infection on pig farms and the swine industry is significant, as the virus causes a high morbidity rate, especially in nursery pigs. This results in economic losses due to decreased growth in younger pigs or increased mortality. PPIV-1 not only leads to economic losses in pig production but also has an important as zoonotic potential virus (1, 10, 14). PPIV-1 can co-infect with Swine Influenza Virus (SIV) and Porcine Reproductive and Respiratory Syndrome Virus (PRRSV), suggesting that the virus may be associated with a complex respiratory disease in pigs from multiple etiology, namely the porcine respiratory disease complex (PRDC) (6, 13). Although PPIV-1 has been reported in America, Europe, and some Asian countries, this virus has never been reported in pig farms in Thailand and Myanmar. Since there is limited information available regarding the epidemiology and current disease status of PPIV-1 in pig farms in Thailand and Myanmar, this study aimed to conduct a survey and genetically characterize PPIV-1 isolated in pig farms from Thailand and Myanmar.

## Materials and methods

### Sample collection from pig farms

**Figure 1:**
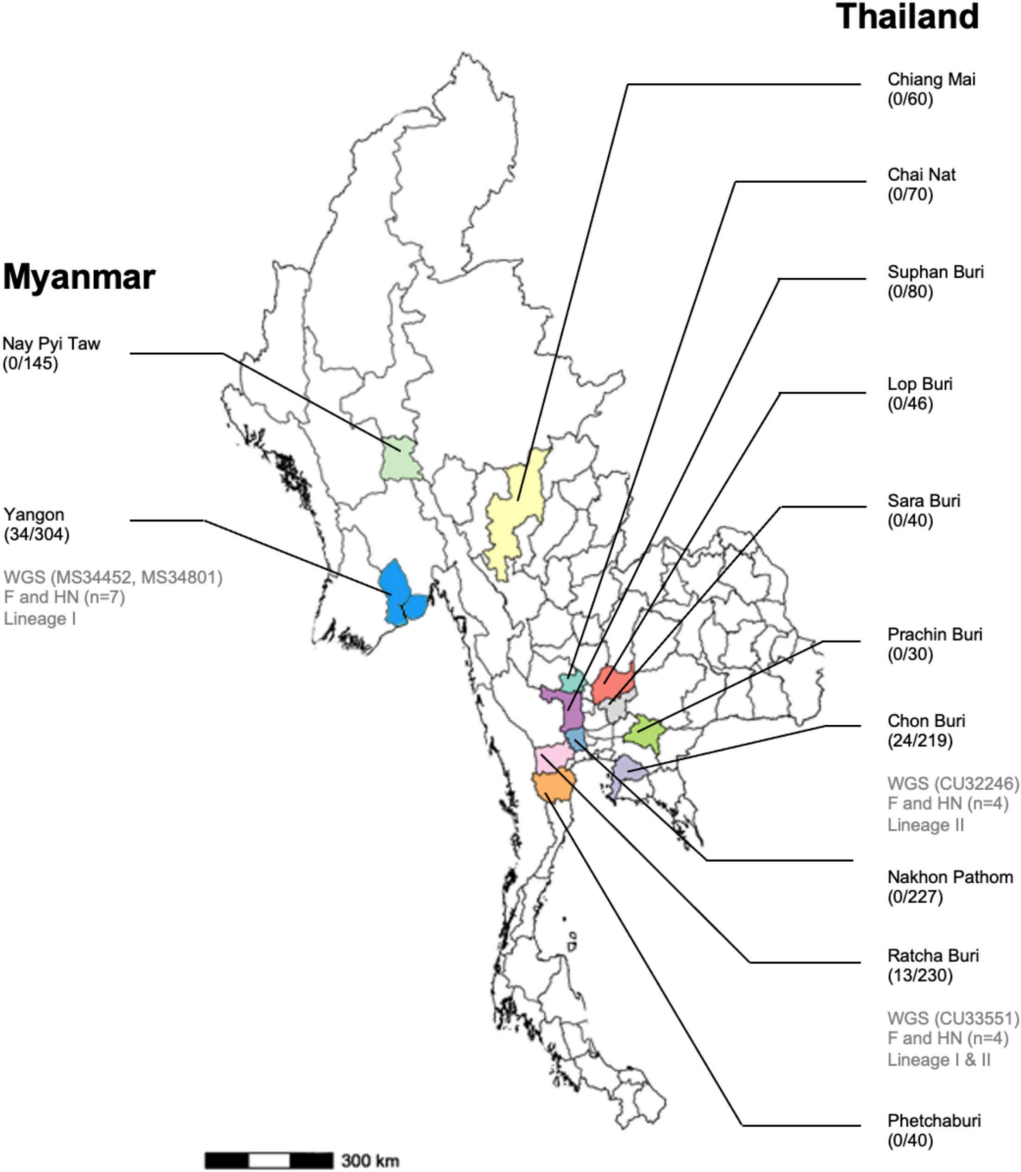
Map of Myanmar and Thailand shows the provinces/states where the samples were collected, the number of PPIV-positive/nasal samples tested, and the number of PPIV-characterized in this study.

In this study, a cross-sectional sample collection was carried out in 26 pig farms in ten provinces of Thailand, including Chainat, Chiangmai, Chonburi, Lopburi, Nakhon Pathom, Prachinburi, Phetchaburi, Ratchaburi, Saraburi, and Suphanburi from January 2022 to December 2023. In Myanmar, a cross-sectional sample collection was conducted in 19 pig farms located in two regions, Yangon and Nay Pyi Taw (Figure 1). The pig farms for sample collection were selected based on the criteria: pig farms being situated in high-density pig production areas and collaboration of pig farm owners. Approximately 10–30 samples were collected from different age groups of pigs per farm on each visit. In total, 1,491 nasal swab samples (NS) were collected from pigs of different ages and pigs with or without respiratory clinical symptoms. The nasal swab sample collected was conducted following the OFFLU guidelines (15). In brief, the swab was inserted into one nostril approximately 2–4 cm deep (depending on the age of the pigs) and gently swabbed using a circular motion for at least 5–10 s. Then, repeat the same procedure in the other nostril with the same swab. Age groups of pigs included the suckling group (≤ 4 weeks), nursery group (> 4–8 weeks), fattening group (> 8–20 weeks), and breeder (Gilt, Sow, Boar). Out of 1,491 samples, 1,042 NS were from Thailand, and 449 NS were from Myanmar. The nasal swab samples were placed in 2 mL viral transport media (MEM; Eagle Minimum Essential Medium, Hyclone™). In 1 liter of MEM, the powder is mixed with distilled water, with 1% Bovine serum albumin (BSA), 250 mg Gentamycin, 10 mL of L-glutamine, and 30 mL of NaHCO_3_. The collected samples were transported at 4°C to the laboratory within 24 h and kept in the freezer until tested, but no later than 4 weeks. The data relating to the collection date, age, sex, breed, and clinical status of the animals were recorded and used to select samples for the viral characterization step. For example, the PPIV-1 positive samples were selected first based on the location of the pig farms, followed by the age of the pigs, and their collection date. The study was carried out under the approval of the Chulalongkorn University Animal Cares and Uses Protocol (CU-VET IACUC 2331099), following the guidelines and regulations of CU-VET IACUC for all animal procedures. The study in Myanmar was conducted with permission from the Livestock Breeding and Veterinary Department (LBVD), Yangon and Nay Pyi Taw, Myanmar.

### Detection of porcine parainfluenza virus 1

RNA was extracted by aliquoting 200 μL of nasal swab samples and subjected to nucleic acid extraction using the Gene All^®^ GENTi™ Viral DNA/RNA Extraction Kit (Gene All^®^; Lisbon, Portugal) on a GENTi™^32^ (GeneAll^®^) following the manufacturer’s recommendations. The RNA samples were tested for PPIV-1 using an RT-PCR assay specific to the L gene using a Super-Script III Platinum Taq One-step RT-PCR kit (Invitrogen, CA, United States). The L gene-specific primers (5’-TTTTGGGTTCAAGAGATTCTT-3′ and 5′- ACCTTTTGGTCTATG-TATAAAGA-3′), were used to detect PPIV-1, as described previously (1). In detail, the One-step RT-PCR reaction mixture included 3 μL of RNA, 12.5 μL of 2X SSIII buffer, 0.5 μM of each forward and reverse primer, and 1 U of Superscript III RT-Platinum Taq Polymerase and nuclease-free water to a final volume of 25 μL. The thermal cycling conditions contained at 55°C for 30 min for the cDNA synthesis step, at 94°C for 2 min for the initial denaturation step, and 35 cycles of denaturation at 94°C for 30 s, annealing at 47°C for 30 s and extension at 68°C for 1 min and final extension step at 68°C for 5 min. Positive samples for PPIV-1 were anticipated to yield a 154 bp PCR product when analyzed using 1.5% agarose gel electrophoresis. In this study, swine influenza virus (SIV) detection was also carried out in all nasal swab samples using real-time RT-PCR to investigate the co-infection of the virus in pigs (16).

### Genetic characterization of porcine parainfluenza virus 1

In this study, the PPIV-1s were selected for whole genome sequencing (*n* = 4) and F and HN gene sequencing (*n* = 11). The PPIV-1 positive samples were selected based on the location of pig farms, the age of the pigs, their collection date, and the high yield of viral RNA by NanaDrop measurement. The samples were selected so that there was no overlap in location, age, and collection date. The samples were also selected based on the gel electrophoresis result, which showed a high yield of PCR products. Whole genome sequencing was performed by amplification of each gene of the viruses with newly designed primer sets using the Primer 3plus program and primers sets reported previously (Supplementary Table S1). In detail, the One-Step RT-PCR reactions were set up separately for each primer pair. The reaction mixture included 3 μL of RNA, 0.5 μL of each forward (10 μM) and reverse primer (10 μM), 12.5 μL of 2X Reaction Mix, 1 μL of SuperScript™ III RT/Platinum™ Taq Mix (cat no. 12574026) (InvitrogenTM; California, United States), and nuclease-free water to a final volume of 25 μL for each reaction. The amplified PCR products were pooled by mixing 20 uL per PCR reaction of one sample. The pooled PCR product (480 μL) was repeatedly loaded onto a single column to purify by using NucleoSpin^®^ Gel and PCR Cleanup (MACHEREY-NAGEL, Düren, Germany) following the manufacturer’s instructions. The library was prepared using the Oxford Nanopore rapid sequencing kit V14 (SQK-RAD114) in accordance with the rapid sequencing protocol (Oxford Nanopore Technologies). A library was prepared per one round. In detail, 5 μL of the purified PCR product was mixed with 0.5 μL of fragmentation mix (FRA), incubated at 30°C for 2 min, then at 80°C for 2 min, and kept on ice. Then, 0.5 μL of diluted rapid adapter (RAP) was added and incubated at 25°C for 5 min. After that, 15 μL of sequencing buffer (SQB) and 10 μL of loading beads (LB) were added to complete the loading DNA library. The final library (at least 20 ng/μl), measured using a NanoDrop spectrophotometer (Thermo Fisher Scientific), was loaded into the flow cell (FLO-FLG114) R10.4.1 on the MinION Mk1b device and sequenced under MinKNOW software (v24.11.8) for 12 h (Oxford Nanopore Technologies, Oxford, United Kingdom). After sequencing, the qualified reads were converted from “Fast 5” into “Fastq” format by using the GPU version of the Nanopore Guppy basecaller (v6.5.7) tool. After basecalling, the EPI2ME platform (v5.2.3) was used to identify potential references for the sequencing data using wf-alignment workflow setting reference as all parainfluenza virus references in the NCBI database. The reads were aligned to references using Minimap2 (V2.28). The resulting alignments were then polished, and the consensus sequence was refined using Medaka (version 2.0.0). The nucleotide sequences were mapped to the reference (PPIV-1/S119N/Hong Kong/2009).

### Phylogenetic and genetic analyses of porcine parainfluenza virus 1

Phylogenetic and genetic analyses were performed by comparing the nucleotide sequences of Thai PPIV-1 and Myanmar PPIV-1 with those of reference viruses under the genus *Respirovirus* available from the GenBank database, including bovine parainfluenza virus 3, caprine parainfluenza virus 3, human parainfluenza virus 1, human parainfluenza virus 3 and murine respirovirus 1 (Sendai virus). The reference strains of PPIV-1s from different geographical regions were also included. Phylogenetic trees of the whole genome, F, and HN genes of PPIV-1s were performed using MEGA v.10.0 with a maximum likelihood method with the general time reversible gamma model with 1,000 bootstrap replicates. For genetic analysis, the nucleotide sequences and deduced amino acids of PPIV-1s were aligned and compared with those of reference viruses using MegAlign, DNASTAR software v.5.03 (DNASTAR Inc., Wisconsin, United States). An analysis of the multiple sequence alignment (MSA) of nucleotide and amino acids of Thai and Myanmar PPIV-1s and those of reference PPIV-1 strains was performed.

## Results

In this study, we conducted a cross-sectional survey in pig farms located in ten provinces of Thailand (*n* = 26 farms) and two regions in Myanmar (*n* = 19 farms) from January 2022 to December 2023. A total of 1,491 nasal swab samples (1,042 NS in Thailand and 449 NS in Myanmar) were collected and tested for PPIV-1 using L gene-specific RT-PCR. Our results showed that 3.65% (38/1042) and 7.57% (34/449) were PPIV-1 positive in Thailand and Myanmar, respectively (Table 1). In detail, PPIV-1 could be detected in 6 out of 26 (23.07%) tested farms in Thailand and 5 out of 19 (26.32%) tested farms in Myanmar. By location, the PPIV-1 positive samples were highly detected in Chonburi, Thailand (24/219; 10.95%) and Yangon, Myanmar (34/304; 11.18%). PPIV-1s were found in healthy pigs at 4.47% (61/1366) and in symptomatic pigs at 8.80% (11/125). In Thailand, PPIV-1 positive proportions were 3.68% (35/952) in healthy pigs and 3.33% (3/90) in symptomatic pigs, while in Myanmar, PPIV-1 was detected in pigs with respiratory signs at 22.86% (Supplementary Table S2). By age group, PPIV-1 was mainly found in nursery pigs (5–8 weeks) at 8.24% (62/752). By season, the positivity of PPIV-1 was highest in the summer season (28/295; 9.49%), which was comparable to the other two seasons in this study (Supplementary Table S2). In addition, the co-infection with SIV was observed in 10 PPIV-1-positive samples (10/72, 13.89%) (Table 1). It is noted that SIV positivity in nasal swab samples in Thailand and Myanmar was 15.26% (159/1042) and 1.56% (7/449), respectively.

**Table 1:** Description of nasal swab samples collected from pig farms in Thailand and Myanmar in this study.

Province/State	Regions	Farm	Date	Age of pigs^a^	Presence of clinical signs	# Nasal swabs	# PPIV-1 positive (%)
Thailand							
Chainat	Central	A–B	Jun-23	Nursery	Healthy	40	0
			Oct-23	Breeder	Healthy	30	0
Chiangmai	Northern	C	Aug-22	Suckling & Nursery	Healthy	60	0
Chonburi	Eastern	D–H	Mar-22	Nursery & Breeder	Healthy	30	4 (13.33%)
			Nov-22	Suckling & Nursery	Respiratory signs	50	0
			Mar-23	Nursery	Healthy	30	20 (66.7)^b^
			Mar-23	Nursery	Healthy	49	0
			Nov-23	Nursery & Fattening	Healthy	60	0
Lopburi	Central	I	Mar-23	Suckling-Breeder	Healthy	46	0
Nakhon Pathom	Central	J–O	Feb-22	Breeder	Healthy	30	0
			Jun-22	Nursery & Fattening	Healthy	60	0
			Aug-22	Suckling & Fattening	Healthy	35	0
			Sep-22	Fattening	Healthy	50	0
			Sep-22	Fattening	Healthy	32	0
			Oct-22	Breeder	Healthy	20	0
Phetchaburi	Southern	P	Aug-23	Nursery	Healthy	40	0
Prachinburi	Eastern	K	Jul-23	Suckling	Healthy	30	0
Ratchaburi	Central	R–W	Feb-22	Suckling & Fattening	Healthy	30	0
			Aug-22	Nursery	Healthy	30	0
			Oct-22	Suckling & Fattening	Healthy	60	9 (15.0%)^c^
			Dec-22	Suckling & Breeder	Healthy	30	0
			Apr-23	Nursery	Respiratory signs	40	3 (7.50%)^c^
			Sep-23	Nursery	Healthy	40	1 (2.50%)^c^
Saraburi	Central	X	Mar-23	Suckling & Breeder	Healthy	40	1 (2.50%)^d^
Suphanburi	Central	Y–Z	Jul-23	Nursery	Healthy	40	0
			Sep-23	Nursery	Healthy	40	0
						1,042	38 (3.65%)
Myanmar							
Yangon	Southern	AA-NN	Jan-23	Suckling	Healthy	30	0
			Jan-23	Nursery	Healthy	25	10 (40.0%)
			Jan-23	Nursery & Fattening	Respiratory signs	35	8 (22.86%)
			Jan-23	Nursery	Healthy	38	0
			Jan-23	Nursery	Healthy	20	0
			Jan-23	Fattening	Healthy	50	0
			Sep-23	Breeder	Healthy	10	0
			Sep-23	Breeder	Healthy	10	0
			Sep-23	Fattening	Healthy	10	0
			Sep-23	Fattening	Healthy	10	0
			Sep-23	Fattening	Healthy	10	0
			Sep-23	Nursery	Healthy	20	7 (35.0%)^e^
			Sep-23	Nursery	Healthy	20	5 (25.0%)^e^
			Sep-23	Nursery	Healthy	16	4 (25.0%)
Nay Pyi Taw	Central	OO-SS	Jan-23	Fattening	Healthy	30	0
			Jan-23	Fattening	Healthy	25	0
			Jan-23	Suckling & Nursery	Healthy	40	0
			Jan-23	Nursery	Healthy	30	0
			Jan-23	Suckling	Healthy	20	0
						449	34 (7.57%)
Total		45				1,491	72/1491 (4.83%)

a Suckling: ≤ 4 Weeks; Nursery: > 4–8 Weeks; Fattening: > 8–20 Weeks; Breeder: Gilt, Sow, Boar.

b Coinfection of PPIV-1 and SIV in Chonburi (Mar-23); CU32245, CU32247, CU32248, CU32254.

c Coinfection of PPIV-1 and SIV in Ratchaburi (Oct-22); CU30318, (Apr-23); CU32451, (Sep-23); CU33551.

d Coinfection of PPIV-1 and SIV in Saraburi (Mar-23); CU31993.

e Coinfection of PPIV-1 and SIV in Yangon (Sep-23); MS34756, MS34769.

In this study, 15 out of 72 PPIV-1 positive samples were subjected to complete genome sequencing (*n* = 4) and F and HN gene sequencing (*n* = 11). This study successfully sequenced the whole genome of PPIV-1s (*n* = 4) with completed nucleotide sequences of all six major genes. The completed F and HN genes of PPIV-1 of PPIV-1 (*n* = 11) were also successfully sequenced. The nucleotide sequences of the viruses were deposited into the GenBank Database under the accession numbers (PP766830-833, and PP761466-487) (Table 2). Our results showed that the genome sizes of two Thai PPIV-1s are 15,194 bp – 15,312 bp, and those of Myanmar PPIV-1s are 15,384 bp −15,388 bp. The genome structure of the virus contained six major proteins encoding nucleocapsid (N), phosphoprotein (P), matrix (M), fusion (F), hemagglutinin-neuraminidase (HN), and large proteins (L) (3’-N/P/M/F/HN/L-5′). Notably, the lengths of all six major genes of Thai and Myanmar PPIV-1s are identical. However, due to the availability of the 3′ and 5′ nucleotide sequences, the overall genome length differs.

**Table 2:** Description of PPIV-1s characterized in this study.

Sample ID	Province/State	Region	Location	Date	Age^a^	Sequencing	Accession number
CU-28392 N	Chonburi	Eastern	Thailand	Mar-22	8wks	F, HN	PP761466, PP761477
CU-28396 N	Chonburi	Eastern	Thailand	Mar-22	8wks	F, HN	PP761467, PP761478
CU-30316 N	Ratchaburi	Central	Thailand	Oct-22	5wks	F, HN	PP761468, PP761479
CU-30326 N	Ratchaburi	Central	Thailand	Oct-22	6wks	F, HN	PP761469, PP761480
CU-32241 N	Chonburi	Eastern	Thailand	Mar-23	6wks	F, HN	PP761470, PP761481
CU-32246 N	Chonburi	Eastern	Thailand	Mar-23	6wks	WGS	PP766830
CU-32451 N	Ratchaburi	Central	Thailand	Apr-23	5wks	F, HN	PP761471, PP761482
CU-33551 N	Ratchaburi	Central	Thailand	Sep-23	5wks	WGS	PP766831
MS-34452 N	Yangon	Southern	Myanmar	Jan-23	5wks	WGS	PP766832
MS-34473 N	Yangon	Southern	Myanmar	Jan-23	5wks	F, HN	PP761476, PP761483
MS-34489 N	Yangon	Southern	Myanmar	Jan-23	4wks	F, HN	PP761473, PP761484
MS-34495 N	Yangon	Southern	Myanmar	Jan-23	4wks	F, HN	PP761474, PP761485
MS-34752 N	Yangon	Southern	Myanmar	Sep-23	4wks	F, HN	PP761475, PP761486
MS-34773 N	Yangon	Southern	Myanmar	Sep-23	6wks	F, HN	PP761476, PP761487
MS-34801 N	Yangon	Southern	Myanmar	Sep-23	8wks	WGS	PP766833

a Suckling: ≤ 4 Weeks; Nursery: > 4–8 Weeks; Fattening: > 8–20 Weeks; Breeder: Gilt, Sow, Boa.

**Figure 2:**
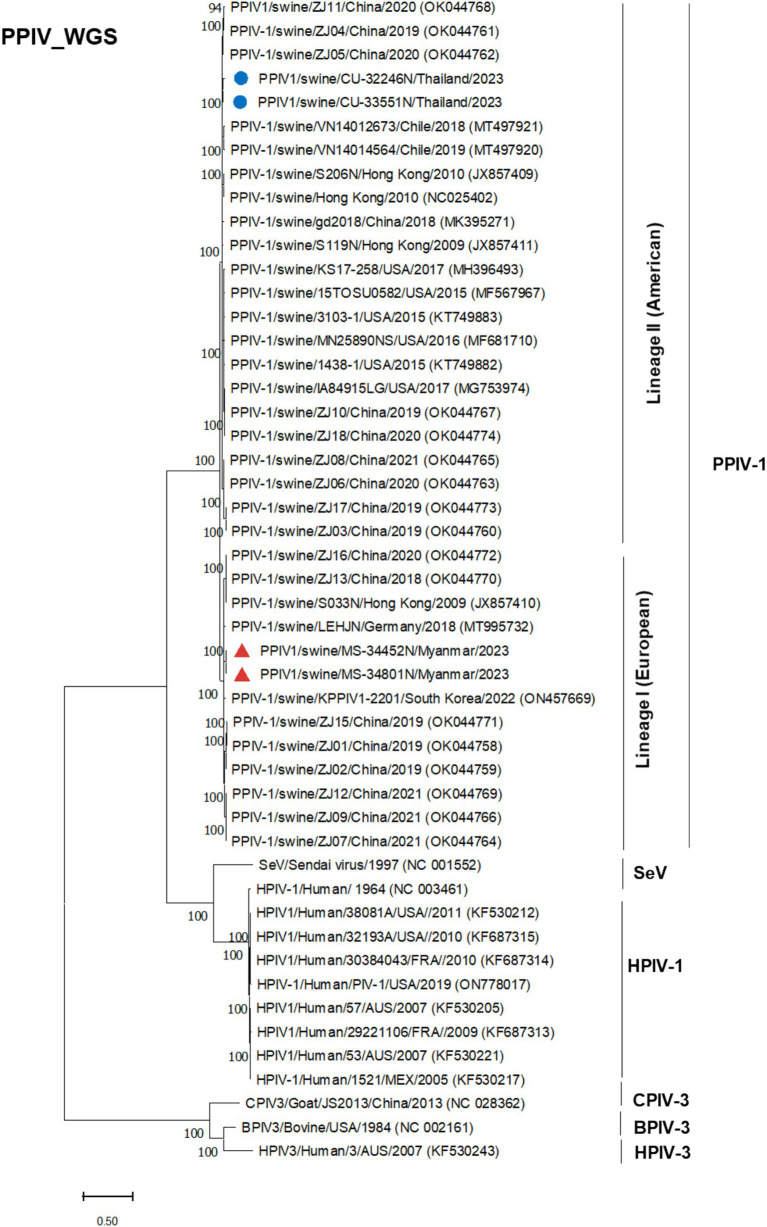
Phylogenetic tree based on whole genome sequences (WGS) of PPIV-1 in this study and reference strains. Blue circles represent Thai PPIV-1, and red triangles represent Myanmar PPIV-1 strains.

**Figure 3:**
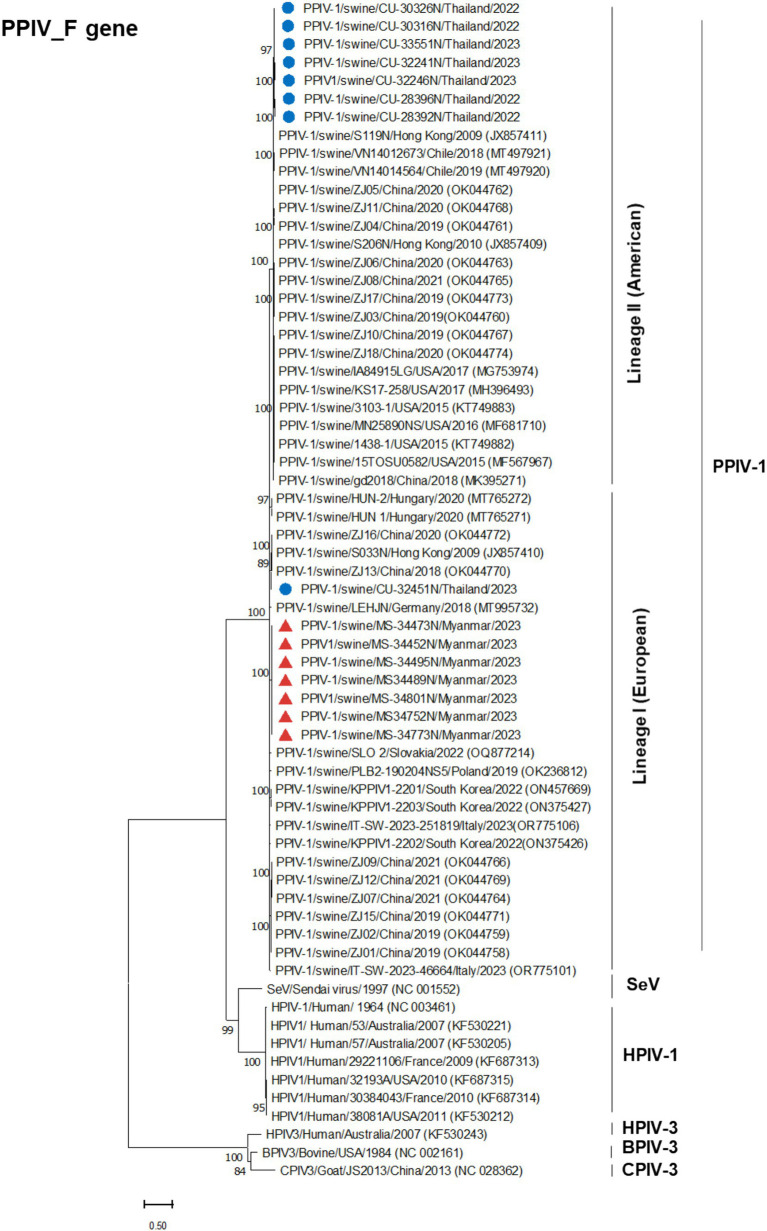
Phylogenetic tree based on F gene of PPIV-1 in this study and reference strains. Blue circles represent Thai PPIV-1, and red triangles represent Myanmar PPIV-1 strains.

**Figure 4:**
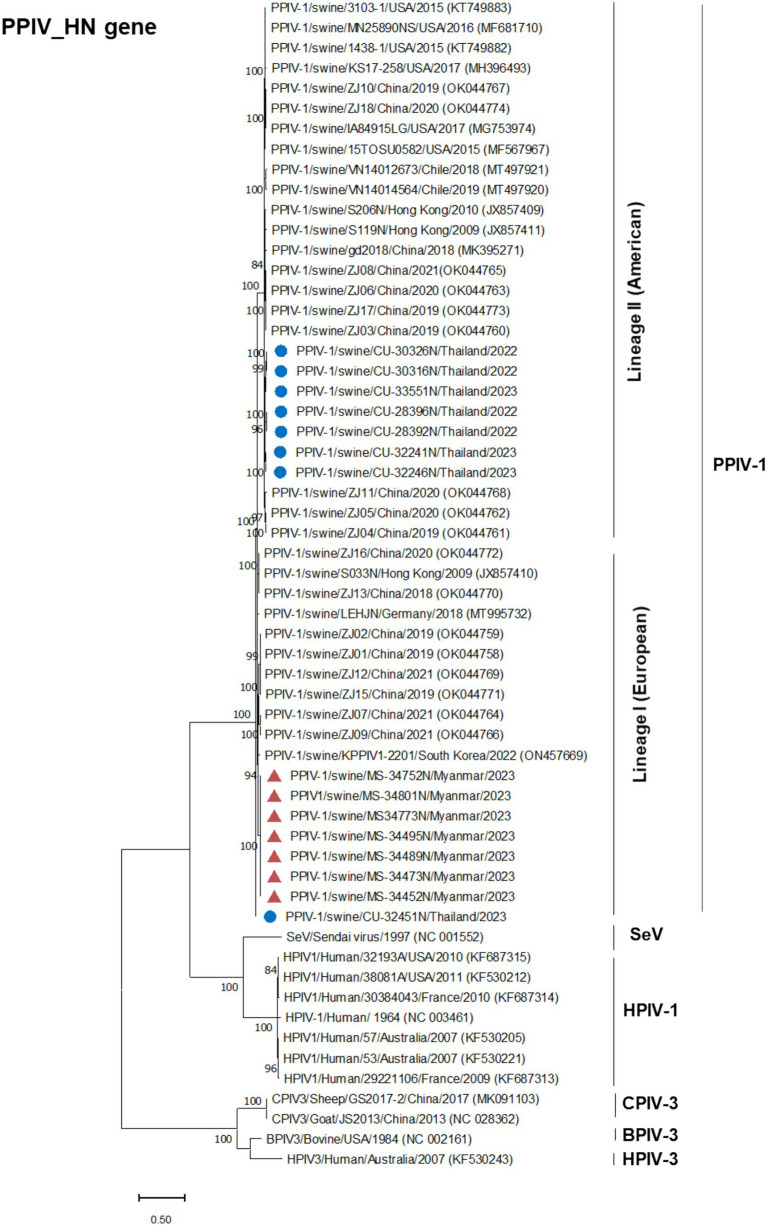
Phylogenetic tree based on HN gene of PPIV-1 in this study and reference strains. Blue circles represent Thai PPIV-1, and red triangles represent Myanmar PPIV-1 strains.

Phylogenetic analysis of the complete genome produced results similar to the F gene analysis, with Thai PPIV-1 being grouped into lineage II (American lineage) and showing close relations to American and Chinese strains. Conversely, Myanmar PPIV-1 was classified into lineage I (European lineage) and was closely related to European, Hong Kong (Chinese), and South Korean strains (Figure 2). It should be noted that both PPIV-1 lineages I and II have been reported in China. Based on the phylogenetic analysis of F genes, PPIV-1 can be classified into two genetic lineages: lineage I (European lineage) and lineage II (American lineage) (5). Our result revealed that 7 out of 7 out of 8 F gene sequences from Thai-PPIV-1 belong to Lineage II, while one sequence (CU32451), belongs to PPIV-1 Lineage I. This finding suggested that PPIV-1 of both lineages (I and II) are circulating in swine in Thailand. On the other hand, Myanmar PPIV-1 (*n* = 7) belonged to lineage I (European lineage) (Figure 3). For the HN gene, the phylogenetic analysis showed that Thai PPIV-1 clustered with lineage II (American lineage) (*n* = 7) and lineage I (European) (*n* = 1), whereas Myanmar PPIV-1 (*n* = 7) grouped with lineage I (European lineage) (Figure 4). The phylogenetic analyses of the N, P, M, and L genes indicated that Thai PPIV-1s also clustered with lineage II (American lineage), while Myanmar PPIV-1 was classified into lineage I (European lineage) (Figures 2, 3; Supplementary Figures S1–S4).

An analysis of the multiple sequence alignment (MSA) of the complete genome of PPIV-1 demonstrated that Thai PPIV-1s exhibited high nucleotide identities with reference PPIV-1s from China (ZJ10, ZJ11), the USA (IA8915), and Chile (VN14012673), showing identities of 95.46–96.91%, with highest % nucleotide identities to China PPIV-1 (ZJ11/2020, 96.91%) (Table 3). For F gene nucleotide comparison, Thai PPIV-1s possessed high nucleotide identity to Hong Kong PPIV-1s (S119N and S206N; 96.50–96.62%), China PPIV-1s (ZJ10 and ZJ 11; 95.99–96.31%) and the USA PPIV-1s (IA84915LG and VN14012673; 95.73–95.40%), respectively. When comparing Thai PPIV-1s with Myanmar PPIV-1s, Hong Kong, Germany, and South Korea strains, the nucleotide identities of the F gene showed 90.03–90.90% (Supplementary Table S3). The nucleotide identities of the HN gene of Thai PPIV-1s showed 94.98–95.74% with Chile, China, Hong Kong (China), and the USA strains (Supplementary Table S4).

**Table 3:** Nucleotide (nt) and amino acid (aa) identities of the complete genome of Thai and Myanmar PPIV-1 with reference PPIV-1 strains.

Virus	Host	Accession number	Lineage	Country	Year	% nt identities (% amino acid identities)						
						WGS (1–15,312)	N (120–1700)	P (1850–3,574)	M (4736–3,693)	F (6615–4,831)	HN (6681–8,411)	L (8574–15,260)
This study												
CU33551	Swine	PP766831	II	Thailand	2023	100.0 (100.0)	100.0 (100.0)	100.0 (100.0)	100.0 (100.0)	100.0 (100.0)	100.0 (100.0)	100.0 (100.0)
CU32246	Swine	PP766830	II	Thailand	2023	96.99 (97.80)	96.33 (99.43)	96.65 (95.82)	96.23 (99.42)	98.24 (98.56)	96.79 (97.57)	97.40 (98.79)
MS34452	Swine	PP766832	I	Myanmar	2023	90.35 (92.81)	91.24 (96.77)	86.72 (85.19)	90.83 (95.39)	90.90 (93.35)	89.85 (94.27)	92.12 (96.60)
MS34801	Swine	PP766833	I	Myanmar	2023	90.33 (92.83)	91.38 (96.95)	86.85 (85.02)	90.95 (95.97)	90.83 (93.35)	89.79 (94.27)	92.04 (96.42)
Reference strains												
S033N	Swine	JX857410.1	I	Hong Kong	2009	90.53 (93.29)	91.08 (96.20)	88.74 (85.71)	90.38 (95.68)	90.03 (93.76)	89.94 (94.44)	92.48 (96.77)
LEHJN	Swine	MT995732.1	I	Germany	2018	90.33 (92.99)	91.90 (96.96)	87.54 (84.67)	90.14 (95.68)	90.13 (93.17)	89.83 (94.10)	92.12 (96.73)
KPPIV-2201	Swine	ON457669.1	I	Korea	2022	90.38 (92.89)	90.58 (96.01)	87.51 (84.32)	89.91 (95.97)	90.49 (92.09)	89.68 (94.10)	92.35 (96.54)
ZJ07	Swine	OK044764.1	I	China	2021	90.60 (93.02)	91.84 (96.41)	87.95 (85.19)	91.08 (95.97)	90.26 (93.35)	89.82 (93.92)	92.47 (96.63)
ZJ09	Swine	OK044766.1	I	China	2021	90.56 (92.98)	91.15 (95.90)	87.94 (85.37)	91.08 (95.97)	90.26 (93.35)	89.87 (93.91)	92.51 (96.68)
S206N	Swine	JX857409.1	II	Hong Kong	2010	96.01 (96.74)	96.07 (97.72)	94.20 (91.46)	94.77 (99.14)	96.62 (96.58)	95.74 (97.05)	97.04 (98.47)
S119N	Swine	JX857411.1	II	Hong Kong	2009	96.01 (96.92)	96.00 (97.72)	94.40 (91.81)	94.57 (98.56)	96.50 (97.48)	95.06 (97.05)	97.13 (98.56)
IA84915LG	Swine	MG753974.1	II	USA	2017	95.46 (96.13)	95.86 (98.67)	93.81 (91.46)	95.30 (98.85)	95.73 (95.50)	95.05 (95.66)	96.43 (98.21)
VN14012673	Swine	MT497921.1	II	Chile	2018	95.64 (96.84)	95.42 (98.37)	94.95 (92.45)	95.10 (99.42)	95.40 (96.04)	94.98 (96.35)	96.64 (98.52)
ZJ10	Swine	OK044767.1	II	China	2019	95.62 (96.29)	96.00 (98.86)	94.00 (91.64)	95.41 (98.85)	95.99 (95.68)	95.36 (95.83)	96.53 (98.25)
ZJ11	Swine	OK044768.1	II	China	2020	96.91 (97.48)	98.72 (99.43)	96.04 (94.08)	96.33 (99.42)	96.31 (97.30)	95.49 (95.49)	97.65 (98.97)

Genetic analysis of the F gene showed that the F gene of PPIV-1s contains 1,674 nucleotides (558 amino acids). The twelve unique amino acid positions were identified, which could be used to classify the lineage or genotype-specific determinants. In particular, six strains of Thai PPIV-1s (lineage II, American lineage) had amino acid substitution, Proline to Serine at position 8 (P8S) of the F protein. Meanwhile, the Thai PPIV-1 strain (CU32451N) contained Leucine (L), which is similar to that of lineage I (European lineage) (Table 4).

**Table 4:** Genetic analysis of the F gene among Thai and Myanmar PPIV-1s from this study and reference PPIV-1 strains.

Virus	Country/Year	Lineage	Accession number	F gene											
				8	103	105	153	282	330	356	367	424	429	553	557
S033N	Hong Kong/2009	I	JX857410.1	L	D	I	M	I	V	K	V	V	L	I	R
LEHJN	Germany/2018	I	MT995732.1	L	A	T	M	I	V	K	V	V	L	I	R
KPPIV-2201	Korea/2022	I	ON457669.1	L	D	I	M	I	V	K	V	V	L	I	R
ZJ07	China/2021	I	OK044764.1	L	D	I	M	I	V	K	V	V	L	I	K
ZJ09	China/2021	I	OK044766.1	L	D	I	M	I	V	K	V	V	L	I	K
MS34452	Myanmar/2023	I	This study	L	D	I	M	I	V	K	V	V	L	I	R
MS34473	Myanmar/2023	I	This study	L	D	I	M	I	V	K	V	V	L	I	R
MS34489	Myanmar/2023	I	This study	L	D	I	M	I	V	K	V	V	L	I	R
MS34495	Myanmar/2023	I	This study	L	D	I	M	I	V	K	V	V	L	I	R
MS34752	Myanmar/2023	I	This study	L	D	I	M	I	V	K	V	V	L	I	R
MS34773	Myanmar/2023	I	This study	L	D	I	M	I	V	K	V	V	L	I	R
MS34801	Myanmar/2023	I	This study	L	D	I	M	I	V	K	V	V	L	I	R
CU32451^a^	Thailand/2023	I	This study	L	I	T	M	I	V	K	V	V	L	I	R
S206N	Hong Kong/2010	II	JX857409.1	P	N	N	I	V	I	Q	I	I	F	T	G
S119N	Hong Kong/2009	II	JX857411.1	T	N	N	I	V	I	Q	I	I	F	T	G
IA84915LG	USA/2017	II	MG753974.1	P	N	N	I	V	I	Q	I	I	F	T	G
VN14012673	Chile/2018	II	MT497921.1	P	N	N	I	V	I	Q	I	I	F	T	G
ZJ10	China/2019	II	OK044767.1	P	N	N	I	V	I	Q	I	I	F	T	G
ZJ11	China/2020	II	OK044768.1	P	N	D	I	V	I	Q	I	I	F	T	G
CU28392	Thailand/2022	II	This study	S	N	N	I	V	I	Q	I	I	F	T	G
CU28396	Thailand/2022	II	This study	S	N	N	I	V	I	Q	I	I	F	T	G
CU30316	Thailand/2022	II	This study	S	N	N	I	V	I	Q	I	I	F	T	G
CU30326	Thailand/2022	II	This study	S	N	N	I	V	I	Q	I	I	F	T	G
CU32241	Thailand/2023	II	This study	S	N	N	I	V	I	Q	I	I	F	T	G
CU32246	Thailand/2023	II	This study	S	N	N	I	V	I	Q	I	I	F	T	G
CU33551	Thailand/2023	II	This study	P	N	N	I	V	I	Q	I	I	F	T	G

a CU32451 is classified as PPIV-lineage I based on F gene phylogenetic analysis.

## Discussion

This study is the first to report and genetically analyze Porcine Parainfluenza Virus 1 (PPIV-1) in pig farms in Thailand and Myanmar. Our study showed that the positivity of PPIV-1s was 4.83% (72/1491), with 3.65% (38/1042) in Thailand and 7.57% (34/449) in Myanmar, respectively. In this study, PPIV-1s were detected in healthy pigs and pigs with clinical signs at 4.47% (61/1366) and 8.80% (11/125), respectively. Our findings are comparable to those of the previous study in Hong Kong (China) (1). However, a higher positive proportion of PPIV-1 has been reported in Germany, Netherlands, Poland, South Korea, and the USA (6, 10, 11, 13). Similarly, in Myanmar, PPIV-1 was detected in pigs with respiratory signs at 22.86% (Supplementary Table S2). By age groups (suckling, nursery, fattening, and breeder), our findings showed that PPIV-1 was mostly detected in younger pigs (nursery and suckling pigs).

In the USA, a study reported a high PPIV-1 positive (43.3%) in samples submitted to a veterinary diagnostic laboratory (2). In Chile, samples from a swine influenza surveillance program of pigs 3–11 weeks of age with clinical symptoms were tested, and PPIV-1 was found to be 18.9% (7). In Europe, PPIV-1 was detected in respiratory samples of pigs in Germany and Netherlands (31.4%) (6), and Poland (76.7%) (13). In Italy, a study showed that 1.55% of pig samples tested were positive for PPIV-1 (12). In Asia, a survey in South Korea, PPIV-1 was detected in oral fluid samples (71.4%), suggesting PPIV-1 was widely distributed in Korean pig farms (11). In China, a study of PPIV-1 detection showed that 64.8% (201/310) of nasal swab samples were PPIV-1 positive (17).

In this study, the discrepancy in the occurrence of PPIV-1 compared to previous reports may be due to differences in the sampling strategy, type of samples, the clinical status of the animals, and the detection method used to detect the viruses. Our results showed that PPIV-1 was most commonly found in nursery pigs, consistent with previous studies (2, 11). PPIV-1 positivity was higher in nursery pigs than in suckling pigs and breeders. We also found that PPIV-1 can co-infect with SIV. Co-infection of PPIV-1 with SIV was observed in 13.89% of PPIV-1 positive samples (10/72). Similarly, the studies in Chile, Germany, and Poland showed co-infection between PPIV-1 and SIV (7, 13, 18). A previous study in Italy reported co-infection among PPIV-1, SIV, and PRRSV (12). However, the severity of clinical signs and the role of PPIV-1 as a primary or secondary pathogen in co-infected animals need further investigation.

Currently (as of April 2024), only 29 whole genome sequences of PPIV-1 are available in the GenBank database. Our result expands the PPIV-1 genomic information in the nucleotide database. This study has provided four complete genomes and eleven F and HN gene sequences of PPIV-1 from Thailand and Myanmar. The complete genome of Thai PPIV-1 (*n* = 2) and Myanmar PPIV-1 (*n* = 2), as well as F and HN gene sequences of Thai PPIV-1 (*n* = 6) and Myanmar PPIV-1 (*n* = 5) were deposited in the database. Based on the phylogenetic analysis of the complete genome, Thai PPIV-1s (*n* = 2) were grouped with lineage II of PPIV-1 (American lineage) and were closely related to American and Chinese strains. On the other hand, Myanmar PPIV-1s (*n* = 2) belonged to lineage I (European lineage) and were closely related to Hong Kong (China), German, and South Korean strains. These findings supported a high genetic diversity of PPIV-1 in the Southeast Asia region, as in the previous report. For the F gene, the phylogenetic analysis showed that Thai PPIV-1s (*n* = 7) were grouped with lineage II (American lineage) except for one Thai PPIV-1 strain (CU32451) (*n* = 1), which is closely related to the lineage I (European lineage), the prototype strain from Hong Kong (China) (S033N; JX857410.1). It should be noted that heterosubtypic recombination events between PPIV-1 lineage I and II had been observed in Chinese PPIV-1 (19). However, the recombination of Thai-PPIV-1 (CU32451) requires further investigation through whole genome analysis sequencing. On the other hand, Myanmar PPIV-1s (*n* = 7) belonged to lineage I (European lineage) and were closely related to Hong Kong (China), German, and South Korean strains. Genetic analysis of the F gene indicated that twelve distinct amino acid positions are specific to a particular lineage. For example, Myanmar PPIV-1 has the same amino acid pattern as PPIV-1 of lineage I, whereas those of the Thai PPIV-1 strains, except CU32451, are similar to PPIV-1 of lineage II. The differences in nucleotides that correspond to unique amino acid positions among lineages can be utilized to develop diagnostic tests for lineage identification in the future.

Due to the global trade of breeding pigs and pig-derived products, pigs can be carriers of transboundary emerging diseases, such as PPIV-1 (20). It is important to note that the pig industry and its products in Thailand heavily depend on imports and exports from the U.S. and European countries. Meanwhile, medium and small-scale pig farms in Myanmar primarily source their livestock through cross-border trade. This reliance can contribute to variations in the PPIV-1 lineage across the region. Hence, it is crucial to genetically characterize these viruses from various geographical regions to determine the origin of PPIV-1s. It is also important to monitor the introduction of PPIV-1 into Thailand and Myanmar.

In conclusion, this study is the first to report and genetically characterize the complete genome of PPIV-1s in pig farms located in Thailand and Myanmar. Phylogenetic analysis showed that Thai PPIV-1s are closely related to PPIV-1s from China and the USA. On the other hand, Myanmar PPIV-1s are closely related to PPIV-1s from Hong Kong (China), Germany, and South Korea. This research provides current disease status and genetic diversity information on PPIV-1 in pig farms located in Thailand and Myanmar. The surveillance and molecular epidemiology of PPIV-1 should be further investigated on a larger scale to monitor the geographical distribution, evolution, and potential zoonotic transmission of PPIV-1.

## Data Availability

The authors declare that the data supporting the findings of this study are available in Supplementary Tables. The nucleotide sequence data that support the findings of this study are openly available in the GenBank database at https://www.ncbi.nlm.nih.gov/genbank/, under accession numbers # PP766830-833, PP761466-487.
