# Supplementary material for: Genetic characterization of porcine parainfluenza virus 1 (PPIV-1) in pig farms: first report of PPIV-1 in Thailand and Myanmar
Source: Front Vet Sci. 2025 Feb 19;12:1435920. doi: 10.3389/fvets.2025.1435920 (PMC11880941; doi:10.3389/fvets.2025.1435920)

Supplement Table 1. Nucleotide sequences of primers used for PPIV-1 detection and sequencing in this study

Primer name	Forward (5'-3')	Primer name	Reward (5'-3')	Position *	Gene	Product size	Reference
Primer detection							
Forward	TTTTGGGTTCAAGAGATTCTT	Reverse	ACCTTTTGGTCTATGTATAAAGA		L	154	Lau et al., 2013
Primer sequencing							
PPIV-1F	ACCAAACAAGAGAAAAGAC	PPIV-565R	TTTTGTCCYTGAAGTAATG	1-565		564	This study
PPIV-N-120F	ATGGCAGGGTTRTTAAGTGTC	PPIV-N-984R	GATTAGATAATGTRAGTGCTGCC	120-984	N	864	This study
PPIV-N-615F	TGTTTAGGAGCAYTGATAGTCCA	PPIV-N-1564R	TCTGCAGCGGATACTTCATC	615-1564	N	949	This study
PPIV-1292F	AGACAGGCTCAAACATCATCTTGC	PPIV-2347R	TCTCCTCGTATTAGAACTCG	1292-2347	N, P	1055	This study
PPIV-P-1847F	TAAATGGATCAGGATGCCYTC	PPIV-P-2887R	AGAYGGGATTACACCAAGACTCAC	1847-1867	P	1040	This study
PPIV-P-2510F	GAAGAGGTACTTAAGAGRAACAAGCC	PPIV-P-3675R	TGAGTCTTGCACCCTAAGTT	2510-3675	P	1165	This study
PPIV-M-3339F	CAAAYCCTGTCCTTGATGAGAG	PPIV-M-4065R	ACACAATCATCTCACTTGACC	4065-3339	M	726	This study
PPIV-M-3855F	TTACTTGGGTTTTATGAGATACC	PPIV-M-4850R	TTTTGGATAACTTGTACCCTAAG	4850-3855	M	994	This study
PPIV-F-4510F	ATGTTCAGCTYACAGGTGTG	PPIV-F-5443R	TCTCCATTGACATAATCCTGGAG	4150-5443	F	933	This study
PPIV-F-5228F	TGTTGGTGCAATTATYGGGAC	PPIV-F-6064R	GCTATRCAGTTYGCAACTACTCC	5228-6064	F	836	This study
PPIV-5823_F	TATGCYGCTGTACCTGACC	PPIV_6640_R	GTGAGTTACTGTCGTGCACCCT	5823-6640	F	817	This study
PPIV-H-6469F	TTACTTATATGTGTAAYTGGATT	PPIV-H-726R	GATGGTAATCTTACACAGCCATC	6469-7269	HN	800	This study
PPIV-H-7163F	TCCAAGTGGRGAACCATATC	PPIV-H-7924R	ACTAGTYTCCCTTCTGCTCC	7163-7924	HN	761	This study
PPIV_7774_F	GCAACAAGTTCATCTCTAGTTCATG	PPIV_8627_R	GCATTCAGGGTATAGTATATCACTTG	7774-8627	HN, L	853	This study
PPIV-L-8288F	TGTCCACTTTGATAGAGGTTATTG	PPIV-L-9327R	CAGCAGACATATTCCATCTACCTTC	8288-9327	L	1039	This study
PPIV-L-8961F	TACTTATGGAGAGATGTGCGTAG	PPIV-L-10164R	ACCAYTCTCCTGTCTCTACATATTG	8961-10164	L	1203	This study
PPIV-L-9745F	AGACCTTATGTGAGTGTCAC	PPIV-L-10852R	ACAGCAGCTAGATGAATTGC	9745-10852	L	1107	This study
PPIV-L-10633	TTGGGTTCAAGAGATTCTTCAACTGG	PPIV-L-11656	GGATTTGGTGACTCTTGGAG	10633-11656	L	1023	This study
PPIV-L-11451F	GCTCTAGCAGACCTCAAAAGG	PPIV-L-12474R	ATTTCATCTGGGTAGCAGTGTC	11451-12474	L	1022	This study
PPIV-L-12111F	TGCAGRTCTGAAGGTGAYAATCC	PPIV-L-13217R	ACCGAAATCCCAGAATCTCTT	12111-13217	L	1106	This study
PPIV-L-12906F	ACAATAGCAGACACCATGTC	PPIV-L-13717R	TTATGTTGGATCTCTTGAGCTAT	12906-13717	L	811	This study
PPIV-L-13510F	TATCTTGTTTAGAGGATCCAAC	PPIV-L-14500R	TACATCTCAGTAGAAGAAGGATTAG	13510-14500	L	990	This study
PPIV-L-14132F	CTGCATTAGTGGCAAAGAAA	PPIV-L-15175R	TTAACAGCTCCAAGTATCTTCAT	14132-15175	L	1062	This study
PPIV-14472F	ACATCTAATCCTTCYTCTACTG	PPIV-15392R	GACAAGAGTTTAAGACATATCA	14472-15392	L	920	This study

*Position based on the strain S119N/Hong Kong 2009 (JX857411.1)

Supplement Table 2. Result of PPIV-1 detection in pig farms in Thailand and Myanmar by location, clinical signs, age group, and season

PPIV-1 detection by location

Location	Region	# farms	# sample	# positive/# sample (%)		
				**Healthy**	**Clinical signs**	
**Thailand**						
Chainat	Central	2	70	0/70	0	
Chiangmai	Northern	1	60	0/60	0	
Chonburi	Eastern	5	219	24/169(14.20%)	0/50 (0%)	
Lopburi	Central	1	46	0/46	0	
Nakhon Pathom	Central	6	227	0/227	0	
Phetchaburi	Southern	1	40	0/40	0	
Prachinburi	Eastern	1	30	0/30	0	
Ratchaburi	Central	6	230	10/190 (5.26%)	3/40 (7.50%)	
Saraburi	Central	1	40	1/40 (2.50%)	0	
Suphanburi	Central	2	80	0/80	0	
**Myanmar**						
Yangon	Central	14	304	26/269 (9.67%)	8/35 (22.86%)	
Nay Pyi Taw	Northern	5	145	0/145	0	
Total		45	1491	61/1366 (4.47%)	11/125 (8.80%)	72/1491 (4.83%)

PPIV-1 detection by clinical signs

Location	# farms	# sample	# positive/# sample (%)		
			**Healthy**	**Clinical signs**	
**Thailand**	26	1042	35/952 (3.68%)	3/90 (3.33%)	
**Myanmar**	19	449	26/414 (6.28%)	8/35 (22.86%)	
Total	45	1491	61/1366 (4.47%)	11/125 (8.80%)	72/1491 (4.83%)

PPIV-1 detection by age group

Age group	Age (weeks)	#sample	Health status	# positive/# sample (%)
Suckling	< 4 weeks	202	healthy	9/202 (4.45%)
Nursery	5 – 8 weeks	752	respiratory signs	62/752 (8.24%)
Fattening	9 -20 weeks	286	healthy	0
Breeder	Gilt, sow, boar	251	healthy	1/251 (0.39%)
Total		1491		72/1491 (4.83%)

PPIV-1 detection by season

Season	Duration	# sample	# positive/# sample (%)
Winter	November-January	483	18/483 (3.73%)
Summer	February-May	295	28/295 (9.49%)
Rainy	June-October	713	26/713 (3.65%)
Total		1491	72/1491 (4.83%)

Supplement Table 3. Nucleotide (nt) and amino acid (aa) identities of F gene of Thai and Myanmar PPIV-1 with reference PPIV-1 strains

	CU33551	CU32246	CU28392	CU28396	CU30316	CU30326	CU32241	CU32451	MS34452	MS34801	MS34473	MS34489	MS34495	MS34752	MS34773	S033N	PIV-2201	LEHJN	ZJ07	ZJ09	S206N	S119N	ZJ10	ZJ11	84915LG	14012673
CU33551		98.6	98.7	98.7	98.7	98.7	98.2	93.0	93.3	93.3	93.3	93.3	93.3	93.3	93.3	93.8	92.1	93.2	93.3	93.3	96.6	97.5	95.7	97.3	95.5	96.0
CU32246	98.2		98.4	98.4	98.0	98.0	99.6	92.4	92.8	92.8	92.8	92.8	92.8	92.8	92.8	93.2	91.7	92.6	92.8	92.8	96.4	96.9	94.6	96.2	94.4	95.3
CU28392	97.4	97.6		100	98.2	98.2	98.0	92.6	93.3	93.3	93.3	93.3	93.3	93.3	93.3	93.8	92.1	93.2	93.3	93.3	96.4	97.1	95.3	96.4	95.1	95.5
CU28396	97.3	97.5	99.9		98.2	98.2	98.0	92.6	93.3	93.3	93.3	93.3	93.3	93.3	93.3	93.8	92.1	93.2	93.3	93.3	96.4	97.1	95.3	96.4	95.1	95.5
CU30316	98.6	97.9	97.3	97.2		100	97.7	92.3	92.6	92.6	92.6	92.6	92.6	92.6	92.6	93.6	91.5	92.4	92.6	92.6	95.7	96.8	94.8	96.6	94.6	95.1
CU30326	98.7	98.0	97.4	97.3	99.8		97.7	92.3	92.6	92.6	92.6	92.6	92.6	92.6	92.6	93.6	91.5	92.4	92.6	92.6	95.7	96.8	94.8	96.6	94.6	95.1
CU32241	98.1	99.6	97.3	97.2	97.8	97.9		92.1	92.4	92.4	92.4	92.4	92.4	92.4	92.4	92.8	91.4	92.3	92.4	92.4	96.0	96.6	94.2	95.9	94.1	95.0
CU32451	90.2	90.1	89.8	89.8	89.9	90.0	89.8		96.0	95.9	96.0	96.0	96.0	96.0	96.0	97.1	94.4	96.2	95.9	95.9	92.6	92.3	91.0	92.4	90.8	91.4
MS34452	90.9	91.0	90.8	90.7	90.8	90.7	90.6	95.2		99.8	100.0	100.0	100.0	100.0	100.0	96.5	96.6	96.6	97.3	97.3	93.3	93.2	91.7	93.0	91.5	92.1
MS34801	90.8	90.83	90.5	90.5	90.7	90.6	90.4	95.1	99.8		99.8	99.8	99.8	99.8	99.8	96.5	96.8	96.6	97.3	97.3	93.3	93.2	91.7	93.2	91.5	92.1
MS34473	90.9	91.0	90.8	90.7	90.8	90.7	90.6	95.2	100.0	99.8		100.0	100.0	100.0	100.0	96.5	96.6	96.6	97.3	97.3	93.3	93.2	91.7	93.0	91.5	92.1
MS34489	90.9	91.0	90.8	90.7	90.8	90.7	90.6	95.2	100.0	99.8	100.0		100.0	100.0	100.0	96.5	96.6	97.3	97.3	97.3	93.3	93.2	91.7	93.0	91.5	92.1
MS34495	90.9	91.0	90.8	90.7	90.8	90.7	90.6	95.2	100.0	99.8	100.0	100		100.0	100.0	96.5	96.6	96.6	97.3	97.3	93.3	93.2	91.7	93.0	91.5	92.1
MS34752	90.8	90.9	90.6	90.5	90.6	90.5	90.5	95.1	99.9	99.9	99.9	99.9	99.9		100.0	96.5	96.6	96.6	97.3	97.3	93.3	93.2	91.7	93.0	91.5	92.1
MS34773	90.8	90.9	90.6	90.5	90.6	90.5	90.5	95.1	99.9	99.9	99.9	99.9	99.9	100.0		96.5	96.6	96.6	97.3	97.3	93.3	93.2	91.7	93.0	91.5	92.1
S033N	90.0	89.9	89.7	89.7	90.1	90.0	89.6	96.3	95.3	95.4	95.3	95.3	95.3	95.3	95.3		95.0	96.5	97.2	97.2	93.4	93.0	91.9	93.2	91.7	92.3
PIV-2201	90.5	90.2	89.9	89.8	90.2	90.1	89.9	94.5	95.9	95.9	95.9	95.9	95.9	95.8	95.8	94.8		95.9	96.0	96.0	92.3	91.9	90.6	91.9	90.5	91.2
LEHJN	90.1	89.9	89.7	89.6	89.9	89.9	89.8	95.1	95.3	95.2	95.3	95.3	95.3	95.1	95.1	95.7	95.1		97.1	97.1	93.5	93.0	91.7	93.2	91.5	91.5
ZJ07	90.3	90.4	90.0	89.9	90.1	90.0	90.1	95.0	96.0	96.1	96.0	96.0	96.0	96.0	96.0	95.9	96.0	95.7		100.0	93.3	93.3	91.7	92.8	91.5	91.7
ZJ09	90.3	90.4	90.0	89.9	90.1	90.0	90.1	95.0	96.0	96.1	96.0	96.0	96.0	96.0	96.0	95.9	96.0	95.7	100		93.3	93.3	91.7	92.8	91.5	91.7
S206N	96.6	96.7	96.0	95.9	96.4	96.5	96.4	90.5	91.1	91.0	91.1	91.1	91.1	90.9	90.9	90.4	90.5	90.6	90.4	90.4		96.6	94.6	95.7	94.4	95.1
S119N	96.5	96.2	96.0	95.9	96.2	96.2	96.0	89.5	90.4	90.4	90.4	90.4	90.4	90.3	90.3	89.6	90.0	89.9	90.0	90.0	96.2		94.8	96.6	94.6	95.5
ZJ10	96.0	95.79	95.4	95.3	95.8	95.9	95.7	89.7	90.4	90.3	90.4	90.4	90.4	90.2	90.2	89.3	89.9	90.1	89.6	89.6	96.0	95.6		94.6	99.8	93.7
ZJ11	96.3	96.2	95.5	95.5	95.9	96.1	95.9	89.8	90.7	90.5	90.7	90.7	90.7	90.5	90.5	89.6	89.7	89.5	89.4	89.4	95.5	95.6	95.1		94.4	95.5
84915LG	95.7	95.5	95.3	95.2	95.5	95.6	95.4	89.6	90.3	90.2	90.3	90.3	90.3	90.2	90.2	89.3	89.8	90.0	89.5	89.5	95.8	95.3	99.8	94.9		93.5
14012673	95.4	95.4	94.7	94.7	95.1	95.1	95.1	89.1	89.1	89.1	89.1	89.1	89.1	89.0	89.0	88.8	89.2	88.6	88.6	88.6	95.2	94.9	94.5	95.0	94.3	

*Values in the left half of the data represent nucleotide sequence identity, and values in the top right half of the data represent amino acid sequence identity

Supplement Table 4. Nucleotide (nt) and amino acid (aa) identities of HN gene of Thai and Myanmar PPIV-1 with reference PPIV-1 strains

	CU33551	CU32246	CU28392	CU28396	CU30316	CU30326	CU32241	CU32451	MS34452	MS34801	MS34473	MS34489	MS34495	MS34752	MS34773	S033N	PIV-2201	LEHJN	ZJ07	ZJ09	S206N	S119N	ZJ10	ZJ11	84915LG	14012673
CU33551		97.6	96.5	96.4	98.1	98.1	97.4	96.4	94.3	94.3	94.3	94.3	94.3	94.3	94.3	94.4	94.1	94.1	93.9	93.9	97.0	97.0	95.8	95.8	95.7	96.4
CU32246	96.8		97.4	97.2	97.7	97.7	99.8	96.1	93.9	93.9	93.9	93.9	93.9	93.9	93.9	93.9	93.9	93.6	93.8	93.7	97.6	97.6	96.9	96.0	96.7	97.4
CU28392	96.7	97.5		99.8	96.5	96.5	97.2	95.4	92.7	92.7	92.7	92.7	92.7	92.7	92.7	93.2	93.1	92.5	92.9	92.9	97.0	97.0	95.3	95.1	95.1	96.7
CU28396	96.6	97.5	99.9		96.4	96.4	97.0	95.4	92.5	92.5	92.5	92.5	92.5	92.5	92.5	93.1	92.9	92.4	92.7	92.7	96.9	96.9	95.1	95.0	95.0	96.5
CU30316	97.6	96.9	96.5	96.5		100.0	97.6	96.1	93.9	93.9	93.9	93.9	93.9	93.9	93.9	94.1	94.1	93.8	93.6	93.6	97.2	97.0	96.0	95.5	95.8	96.4
CU30326	97.8	97.0	96.7	96.6	99.9		97.6	96.1	93.9	93.9	93.9	93.9	93.9	93.9	93.9	94.1	94.1	93.8	93.6	93.6	97.2	97.0	96.0	95.5	95.8	96.4
CU32241	96.7	99.8	97.4	97.3	96.8	96.9		95.9	93.8	93.8	93.8	93.8	93.8	93.8	93.8	93.8	93.8	93.4	93.6	93.6	97.4	97.4	96.7	95.8	96.5	97.2
CU32451	92.0	92.1	91.6	91.6	91.4	91.3	91.8		97.3	97.3	97.3	97.3	97.3	97.3	97.3	98.3	97.8	98.1	97.3	97.3	96.1	96.1	95.6	96.1	95.6	95.9
MS34452	89.8	90.0	89.8	89.7	89.3	89.4	89.8	95.5		100.0	100.0	100.0	99.8	100.0	100.0	96.5	97.4	96.2	97.2	97.2	93.6	93.6	93.4	93.2	93.2	93.2
MS34801	89.8	90.0	89.7	89.7	89.3	89.4	89.8	95.3	99.8		100.0	100.0	99.8	100.0	100.0	96.5	97.4	96.2	97.2	97.2	93.6	93.6	93.4	93.2	93.2	93.2
MS34473	89.8	90.0	89.8	89.7	89.3	89.4	89.8	95.5	100.0	99.8		100.0	99.8	100.0	100.0	96.5	97.4	96.2	97.2	97.2	93.6	93.6	93.4	93.2	93.2	93.2
MS34489	89.8	90.0	89.8	89.7	89.3	89.4	89.8	95.5	100.0	99.8	100.0		99.8	100.0	100.0	96.5	97.4	96.2	97.2	97.2	93.6	93.6	93.4	93.2	93.2	93.2
MS34495	89.9	90.0	89.8	89.7	89.4	89.4	89.8	95.5	99.9	99.8	99.9	99.9		99.8	99.8	96.4	97.2	96.0	97.0	97.0	93.6	93.6	93.4	93.2	93.2	93.2
MS34752	89.8	90.0	89.7	89.7	89.3	89.4	89.8	95.3	99.8	100.0	99.8	99.8	99.8		100.0	96.5	97.4	96.2	97.2	97.2	93.6	93.6	93.4	93.2	93.2	93.2
MS34773	89.8	90.0	89.7	89.7	89.3	89.4	89.8	95.3	99.8	100.0	99.8	99.8	99.8	100.0		96.5	97.4	96.2	97.2	97.2	93.6	93.6	93.4	93.2	93.2	93.2
S033N	89.9	90.1	90.2	90.1	89.9	89.8	89.9	96.8	95.4	95.3	95.4	95.4	95.4	95.3	95.3		96.5	97.0	96.5	96.5	93.8	93.8	93.6	93.9	93.4	93.4
PIV-2201	89.7	90.5	89.8	89.8	89.7	89.8	90.3	96.2	96.4	96.2	96.4	96.4	96.3	96.2	96.2	95.7		96.4	98.3	98.3	93.4	93.6	93.2	92.9	93.1	93.8
LEHJN	89.8	90.4	89.8	89.7	89.7	89.5	90.2	96.7	95.3	95.1	95.3	95.3	95.2	95.1	95.1	96.2	95.4		96.0	96.0	93.4	93.4	93.6	93.6	93.4	93.1
ZJ07	89.8	90.4	89.9	89.8	89.5	89.5	90.2	95.7	96.2	96.1	96.2	96.2	96.1	96.1	96.1	95.5	97.0	95.2		100.0	93.2	93.2	93.4	92.9	93.2	93.2
ZJ09	89.9	90.5	90.0	89.9	89.6	89.6	90.3	95.7	96.2	96.2	96.2	96.2	96.2	96.2	96.2	95.6	97.0	95.3	99.9		93.2	93.2	93.4	92.9	93.2	93.2
S206N	95.7	96.4	96.2	96.2	95.5	95.6	96.3	92.0	89.8	89.7	89.8	89.8	89.8	89.7	89.7	90.0	89.8	90.0	90.0	90.1		98.6	96.9	96.2	96.7	97.4
S119N	95.1	95.3	95.6	95.5	95.2	95.3	95.2	91.3	89.4	89.3	89.4	89.4	89.4	89.3	89.3	89.7	89.6	89.7	89.6	89.6	96.9		96.9	96.2	96.7	97.4
ZJ10	95.4	96.0	95.6	95.6	95.4	95.6	95.9	91.8	89.7	89.6	89.7	89.7	89.7	89.6	89.6	90.2	89.6	90.0	90.0	90.0	96.8	95.9		95.1	99.8	96.4
ZJ11	95.5	95.7	95.4	95.4	94.9	95.0	95.5	92.5	90.1	90.1	90.1	90.1	90.1	90.1	90.1	90.3	89.9	90.5	90.3	90.2	96.1	95.2	95.7		95.0	95.5
84915LG	95.1	95.8	95.3	95.2	95.1	95.2	95.7	91.5	89.3	89.2	89.3	89.3	89.3	89.2	89.2	89.8	89.4	89.6	89.8	89.8	96.5	95.6	99.7	95.4		96.2
14012673	95.0	95.9	95.6	95.6	94.9	95.0	95.7	91.5	89.2	89.2	89.2	89.2	89.2	89.2	89.2	89.4	89.3	89.2	89.6	100	96.1	94.9	95.7	94.9	95.4	

*Values in the left half of the data represent nucleotide sequence identity, and values in the top right half of the data represent amino acid sequence identity

Supplement Figure 1. Phylogenetic tree based on N gene of PPIV-1 in this study and reference strains. Blue circles represent Thai PPIV-1, and red triangles represent Myanmar PPIV-1 strains.

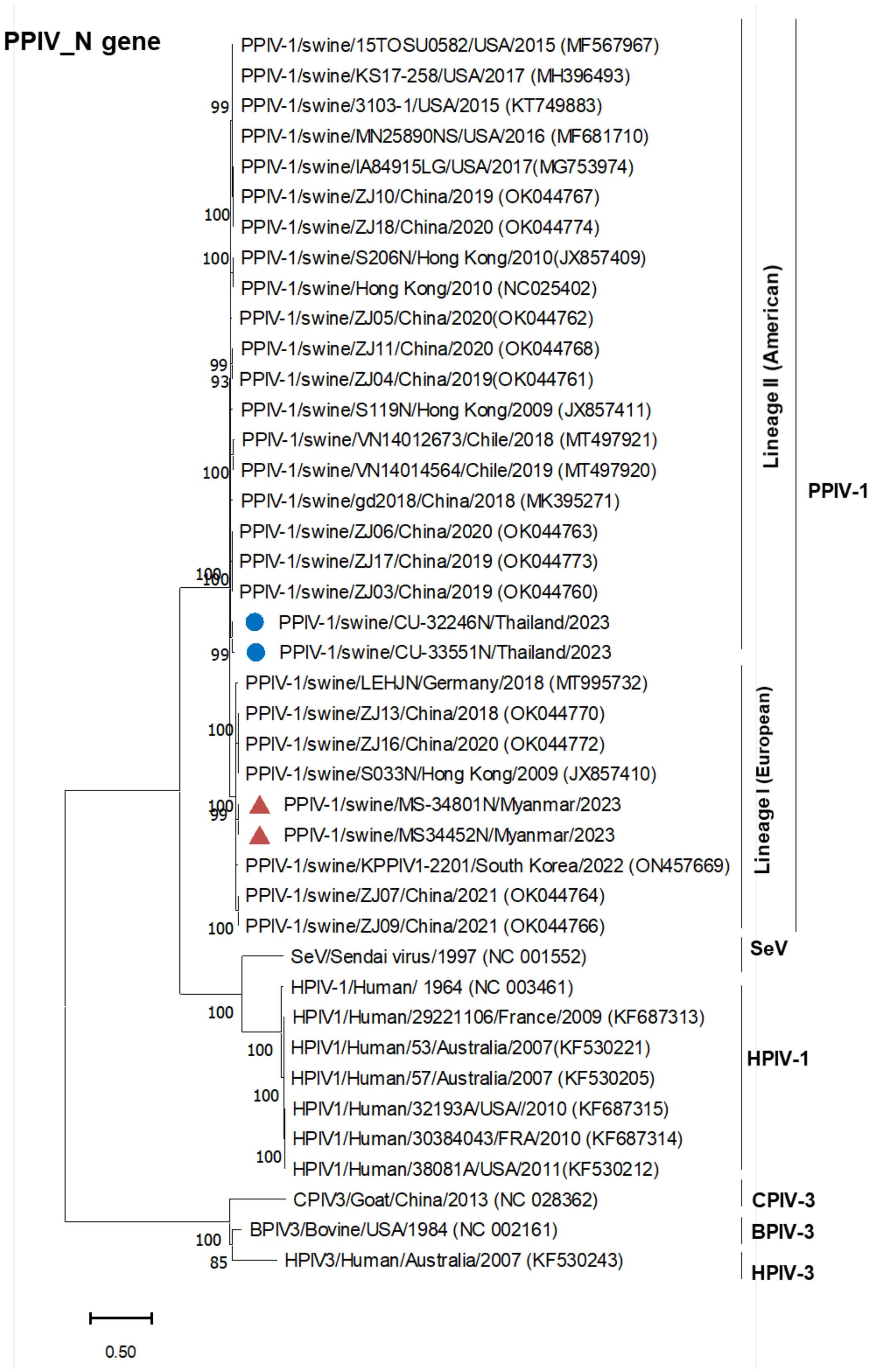

Supplement Figure 2. Phylogenetic tree based on P gene of PPIV-1 in this study and reference strains. Blue circles represent Thai PPIV-1, and red triangles represent Myanmar PPIV-1 strains.

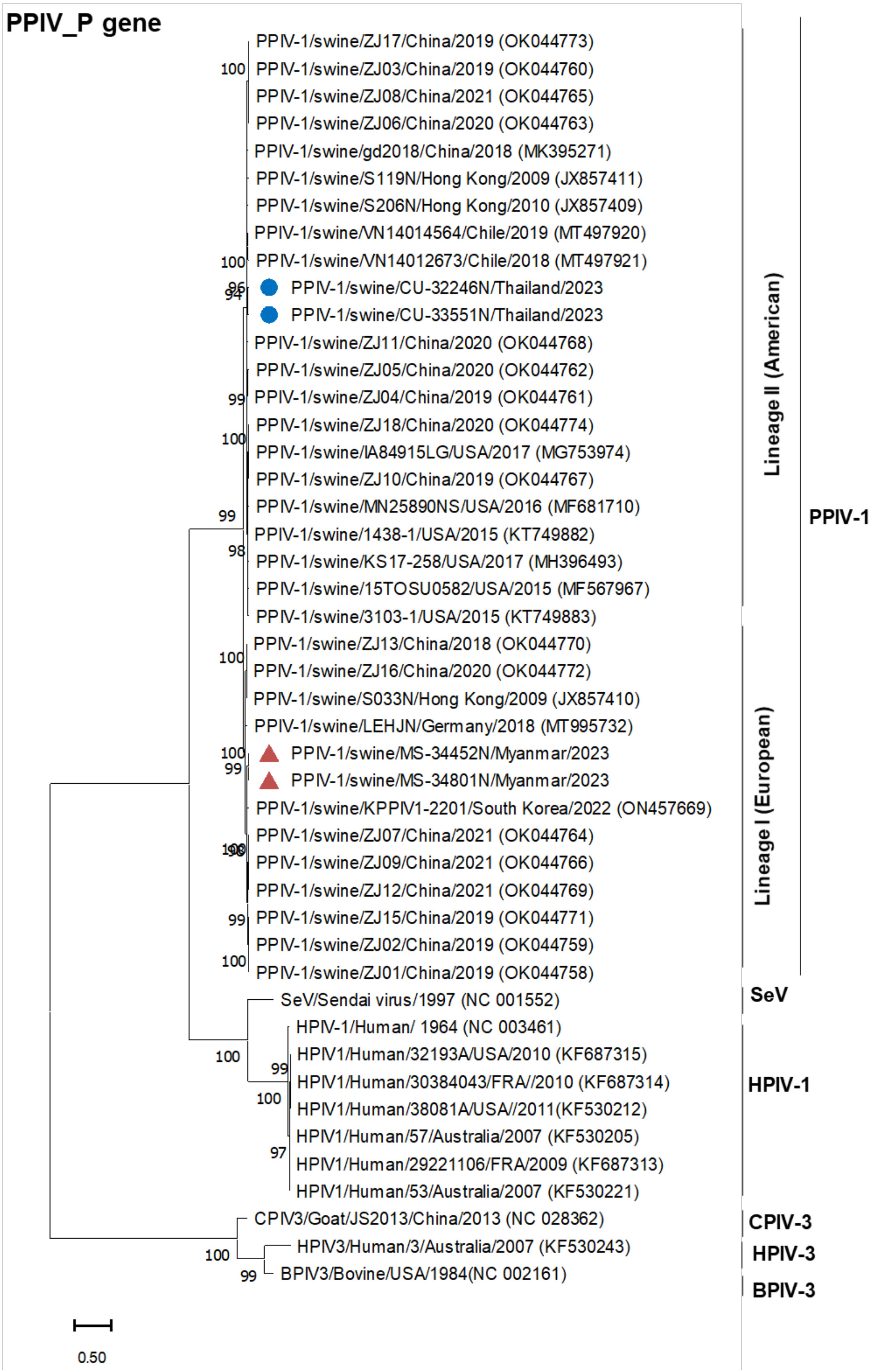

Supplement Figure 3. Phylogenetic tree based on M gene of PPIV-1 in this study and reference strains. Blue circles represent Thai PPIV-1, and red triangles represent Myanmar PPIV-1 strains.

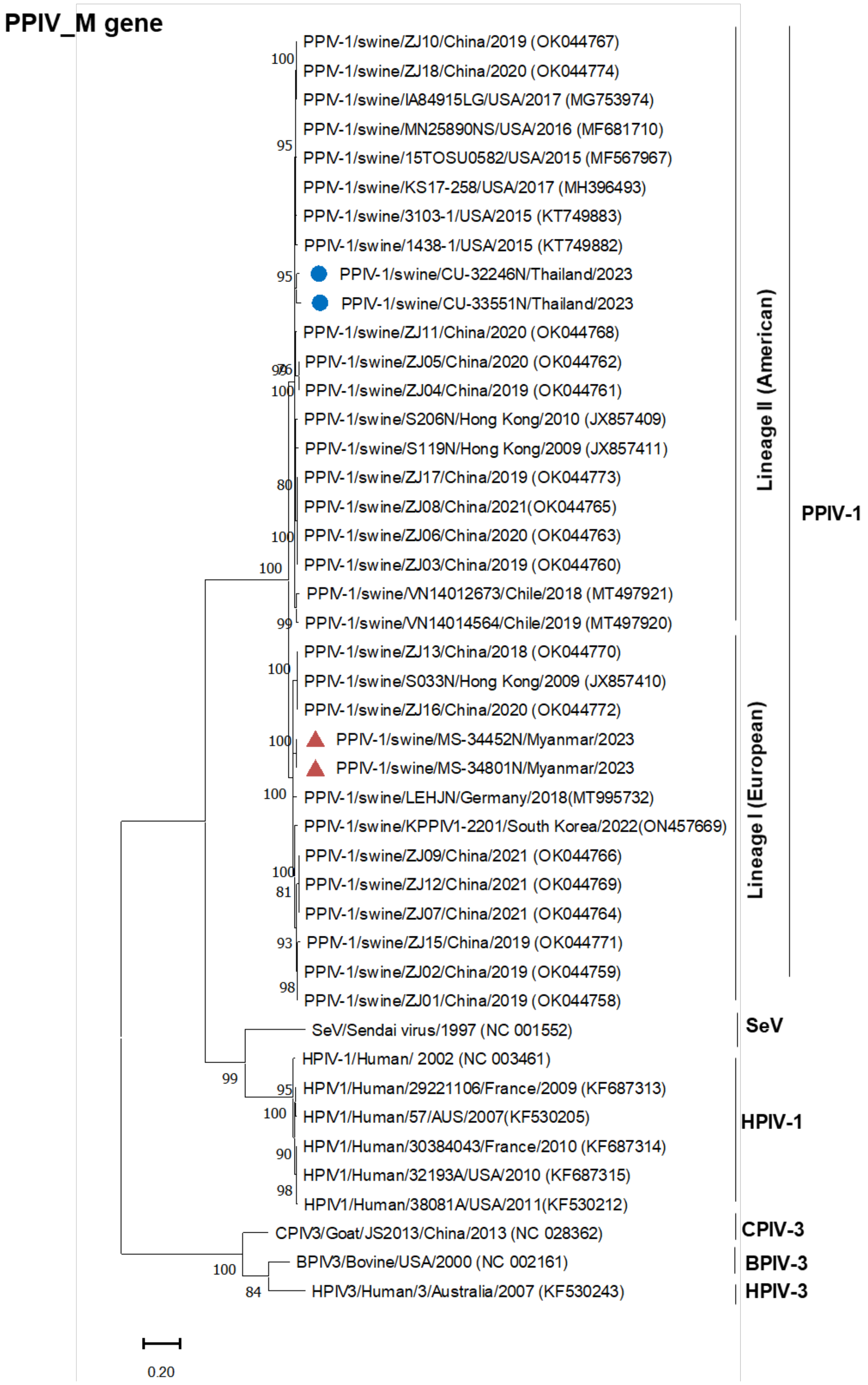

Supplement Figure 4. Phylogenetic tree based on L gene of PPIV-1 in this study and reference strains. Blue circles represent Thai PPIV-1, and red triangles represent Myanmar PPIV-1 strains.

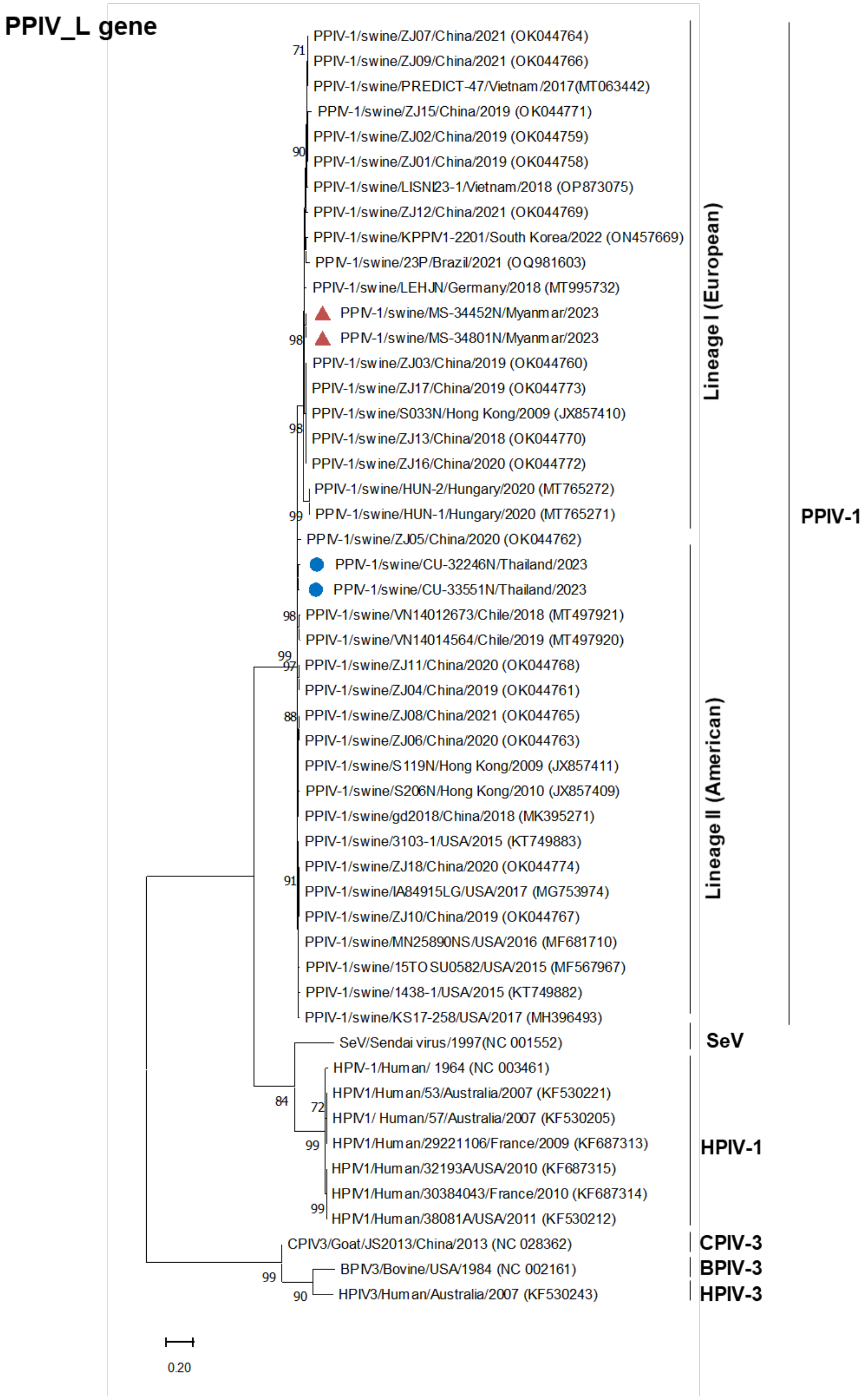

Supplement: Supplementary file 1 [file Data_Sheet_1.pdf]
